# A public health approach to addressing and preventing misdiagnosis in the scale-up of HIV rapid testing programmes

**DOI:** 10.7448/IAS.20.7.22190

**Published:** 2017-08-29

**Authors:** Cheryl C. Johnson, Shona Dalal, Rachel Baggaley, Miriam Taegtmeyer

**Affiliations:** ^a^ Department of HIV, World Health Organization, Geneva, Switzerland; ^b^ London School of Hygiene and Tropical Medicine, Department of Clinical Research, London, UK; ^c^ Liverpool School of Tropical Medicine, Liverpool, UK

**Keywords:** HIV, diagnostic, rapid diagnostic test, test, quality, misdiagnosis, misclassification

## Introduction

The global impact of the scale-up HIV testing and treatment has been impressive. In 2015, approximately 60% of people with HIV worldwide were aware of their status [1]. As a result by the end of 2015, 17 million people with HIV were on treatment, and global treatment coverage reached 46% [1]. HIV testing and treatment have reduced AIDS-related deaths by 43% since 2003 [1,2]. In order to further increase impact and improve health outcomes, in 2016 the World Health Organization (WHO) recommended antiretroviral therapy (ART) for all people with HIV regardless of disease status [3]. These calls to continue scale-up of testing and treatment and to achieve the United Nation’s (UN) “90-90-90” targets remain a global priority. Achieving the “first 90” by reaching people with HIV who have yet to be diagnosed, and linking them to treatment as early as possible, is a critical first step.

Degrees of uncertainty exist with all medical testing and diagnoses; in the field of HIV, advances in diagnostic test technology have made testing accurate and reliable. WHO prequalified HIV rapid diagnostic tests all have a sensitivity of ≥99% and specificity ≥98% and are accurate when used correctly in a validated national algorithm. A large number of tests are conducted every year. Although a degree of error and misdiagnosis can be expected, very few cases of false negative and false positive diagnoses have been reported [4–12]. This lack of reporting on testing error and misdiagnoses is not unique to HIV [13–16]. Publication bias and concerns about programme reputation may have contributed to low reporting of misdiagnosis and limit the open discussion required to address errors systematically [16].

To further investigate diagnostic error, determine common causes, and identify potential ways to address misdiagnosis, particularly in resource-limited settings, WHO, Liverpool School of Tropical Medicine and Médecins Sans Frontières (MSF) held a symposium to address the social, public health, human rights, ethical and legal implications of misdiagnosis of HIV status [17]. This special issue of *the Journal of the International AIDS Society* follows this symposium by focusing on the individual and public health implications of HIV misdiagnosis.

## Is HIV misdiagnosis a “real” problem?

Data from a systematic review of 64 studies (most studies identified were conducted in Africa and other resource-limited settings) are included in this special issue and summarize the magnitude of misdiagnosis in these contexts. The review suggests that on average 0.4% (interquartile range (IQR): 0–3.9%) of diagnoses primarily among adults are false negative and 3.1% (IQR: 0.4–5.2%) are false positive [18]. Among people diagnosed with HIV who were enrolled in care and/or on ART, between 0.1% and 6.6% of patients were reported to be truly HIV negative and had been misdiagnosed [18]. The diagnostic errors identified were largely related to human error [18]. Although reported levels of misdiagnosis are low, if current estimates are accurate [18,19], the large volume of tests conducted each year - over 150 million tests in low- and middle-income countries in 2014 alone, 3 million of which were HIV positive [19] - could result in the misdiagnosis of up to 93,000 people per year if left unaddressed.

## What factors and processes contribute to misdiagnosis using rapid tests?

**Figure 1.**
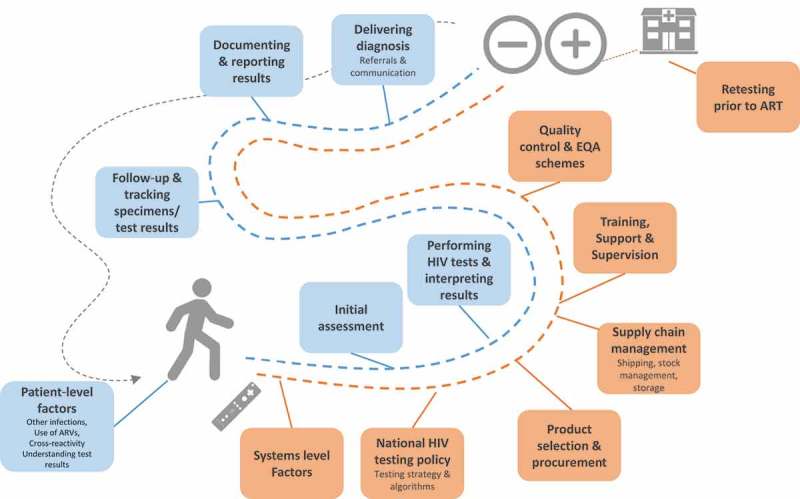
HIV testing continuum and the patient, provider, facility and system-level dimensions of where diagnostic errors and HIV misdiagnosis can occur [20].

HIV misdiagnoses and testing errors are unlikely to be the result of a single cause or underlying factor. Diagnostic errors can occur across multiple steps within the HIV testing continuum, starting from national policy and training, through the supply chain, initial testing and the delivery of a diagnosis, including retesting patients prior to ART initiation as well as inadvertently retesting patients on ART who may re-present for testing erroneously (Figure 1).

The review by Johnson et al. [18] highlighted that the use of suboptimal testing algorithms was a common cause of misdiagnoses in studies reviewed. In this issue, Bock et al. report that by using a first-line assay with poor sensitivity in South Africa, the resulting programmatic sensitivity was as low as 45% (95% confidence interval: 23–48) [21]. Another known contributor to misdiagnosis is the use of a tiebreaker test to rule in HIV infection after discrepant test results, which can cause a high proportion of false positive diagnoses. Although this strategy is known to be inferior to providing patients with an inconclusive status and requesting them to retest in 14 days, many programmes continue to use a tiebreaker out of convenience, the desire to make an immediate diagnosis so that ART can be initiated  and concerns that clients will be lost to follow-up [22].

User and clerical errors at testing sites are also a factor as reported by Khan et al. in this issue [23]. In their study, nearly all of the misdiagnosed patients unnecessarily placed on ART reported that they had at least one HIV-negative test result before they were started on treatment; additionally, two other patients reported that they were never shown their test results despite being given an HIV-positive diagnosis. Authors suggest these cases of misdiagnosis were likely due to administrative error, user error and clients’ circumstantial belief that they were HIV positive [23]. Thus, it is likely that these cases of misdiagnosis could have been prevented if health workers had a clear understanding of how to communicate uncertainty of test results and if procedures for addressing potential misdiagnoses were in place.

Other reported factors, related specifically to false negative diagnoses, included early/acute HIV infection [24] and testing among people on ART (e.g. people who retest without disclosing their ART use) [18]. According to Kufa et al., false negative diagnoses were associated with a reactive HIV-1 Limiting Antigen enzyme immunoassay (LAg EIA) test result (i.e. a marker of early/acute infection), individuals reporting an HIV-positive status and those reporting ART use [24]. Patients on ART who retest may have low levels of detectable HIV antibodies. Two reports in this issue found that 8.5–44% of false negative diagnoses were among people on ART who were retested [21,25]. According to Olaru et al. [25], which sought to investigate the impact of ART on test performance, 8.5% of children with HIV on ART had a false negative test result when using an oral fluid-based rapid diagnostic test (RDT), and those who had been on ART longer and who had higher CD4 counts were more likely to have a negative test.

Understanding the factors contributing to misdiagnosis across specific contexts is critical to developing a public health approach that will be effective in both addressing and preventing misdiagnosis in the scale-up of HIV rapid testing programmes.

## What are the consequences and costs of misdiagnosis?

The importance, and possible consequences, of misdiagnosis should not be underestimated. On an individual level, false positive diagnoses can lead to unnecessary financial expenses, clinical visits and treatment initiation causing physical, emotional and psychosocial harm [12,23,26]. According to an MSF study, many of those misdiagnosed were not identified and re-diagnosed as HIV negative for at least a year during which 10 people were placed on ART: six for treatment, and four (two mother–baby pairs) to prevent vertical transmission [12]. While the majority of those misdiagnosed were pleased to learn they were HIV negative, several were reportedly overwhelmed by the news as their HIV-positive diagnosis had disrupted their lives through stigma, broken relationships and divorce [12]. Additionally, missed opportunities to diagnose HIV due to false negative results continues to cause delays in the initiation of life-saving treatment and contributes to both on-going HIV transmission and HIV-related morbidity in adults, and as reported by Technau et al. in this issue, amongst HIV-exposed infants with inconclusive results [27].

The potential financial and economic cost of false misdiagnoses is likely to be high. False positive diagnoses, as reported in South Africa by Hsiao and colleagues in this issue [28], lead to unnecessary ART costs, even with ≤1% misdiagnoses. Mathematical modelling suggests programmes which do not retest people with an HIV positive diagnosis prior to initiating treatment could spend between US$58,000 and US$225,000 in unnecessary ART costs per year for both low (1%) and high (10%) HIV prevalence settings [29]. Since there is currently no available HIV testing technology validated for testing people on ART, alternative strategies to determine a patient’s true HIV status after they start treatment require the testing of viral reservoirs [30–32]. These strategies are not only complex, unfeasible in many settings and costly, but ill-advised as they could be potentially harmful to patients. Additionally, as many patients are now offered treatment immediately after diagnosis, the occurrence of seroreversion among patients on treatment may become more common, especially among infants and children [25]. Understanding the implications and the best practices to address retesting among people on ART, as well as the potential implications for retesting people on pre-exposure prophylaxis, is an area needing further research that can guide the implementation of practical solutions.

From a public health perspective, misdiagnoses in the context of HIV surveillance may result in under- or overestimations of HIV prevalence and may have particular implications when programme data from RDT are used [33,34]. False negative diagnoses could also lead to further HIV transmission by providing a false sense of security. As many as 70% of new HIV transmissions may be attributable to undiagnosed HIV infection [35], with early/acute infection contributing to 10–50% of new HIV transmissions [36]. Furthermore, misdiagnoses may also undermine public trust in test results as well as trust in health services. Such distrust can be detrimental, as it can be a barrier preventing and delaying individuals from accessing services [37], potentially exacerbating gaps in HIV testing, prevention and treatment coverage.

## Are there additional challenges to addressing misdiagnosis in infants and children?

Sacks et al. [38] note a number of key differences affecting the interpretation and management of test results in infants and children such as vertical transmission dynamics and the natural history and decay of maternal antibodies. The consequences of delayed, false negative and false positive diagnoses, while serious for all ages, are more so for children. Inconclusive results delayed delivery of a final HIV-positive diagnosis; and 17% of infants with HIV with an inconclusive diagnosis died [27]. Olaru et al. note that some children who start ART early in life never develop HIV antibodies to establish a definitive HIV diagnosis [25], making cases where HIV-negative infants are unnecessarily placed on treatment even more challenging to resolve [38]. With these challenges in mind, in order to address and minimize misdiagnosis in infants and children, it is an urgent priority to retain all HIV-exposed infants and children with HIV-negative or inconclusive test results in care until a final diagnosis is ascertained after completion of breastfeeding and for testing to verify the HIV status of any child who has an initial nucleic acid test with detectable results immediately [38].

## How should HIV misdiagnosis be prevented and addressed?

**Figure 2.**
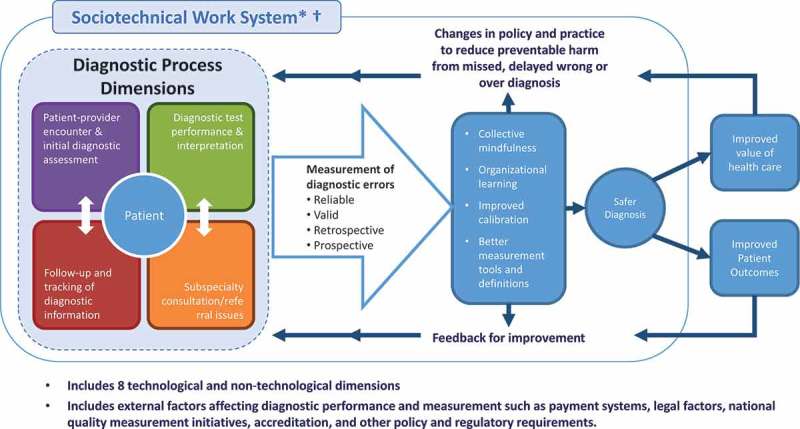
*Safer Dx framework, adapted from Singh and Sittig 2015* [39].

A combination of policy and programmatic approaches will be needed to address and prevent misdiagnoses. As outlined by Singh and Sittig in the “Safer Dx framework” (Figure 2) [39], preventing error and misdiagnosis will require a variety of stakeholders including researchers, health workers, policymakers, programme managers, implementing partners, civil society and patient advocates to develop and implement strategies and tools for measuring and monitoring diagnostic error, as well as to provide feedback and learning to inform the implementation of interventions that minimize misdiagnoses, improve testing quality and result in improved patient outcomes.

First, ensuring that appropriate and quality-assured tests are selected and procured based on a proven testing strategy and validated testing algorithm is critical. A 2015 policy review suggested that fewer than 20% of national HIV testing strategies were in line with WHO recommendations [40]. Revising these strategies to ensure that a sensitive first-line assay and referral of discrepant results for retesting at 14 days are used instead of using a tiebreaker will have a significant impact. Conducting or utilizing findings from testing algorithm validation studies is a key way programmes can reduce the risk of misdiagnosis. Although additional resources may be needed to validate algorithms and replace the use of a tiebreaker test with active follow-up and testing of patients with inconclusive results, this is likely to be a better investment than continuing to deliver potentially incorrect test results which may lead to the unnecessary ART initiation.

Second, retesting prior to ART initiation should be implemented as a routine service and considered the standard of care [17,41]. Despite some concerns about potential costs and feasibility, it is cost-effective [28,29], and can improve testing quality and reduce misdiagnosis. Programmatic reports from Malawi show that since implementing retesting prior to ART, together with retraining testers and introducing new guidelines on supervision, the proportion of patients misdiagnosed has decreased from approximately 7% in 2014 to 1% in 2016 [42].

Third, quality assurance systems are essential to HIV testing services. Training, support and supervision are key for all HIV testing providers, as well as routine external quality assessment schemes which help identify where problems may be occurring and which HIV testing providers and sites might benefit most from additional training and support. Standardized testing registers and logbooks improve reporting systems and, when maintained, can help programmes quickly identify and assess emerging quality issues and take corrective action. Electronic record systems can accelerate the identification of errors and misdiagnoses by quickly gathering and assessing patient-level information and tracking and following-up of those with characteristics linked to misdiagnosis [43]. The assignment of a unique patient identifier so that all of the testing results for the one client can be followed and evaluated over time is critical to identifying potential misdiagnosis.

Many of the studies included in this special issue are examples of how investigating quality issues can lead to improvements in testing services and how combining quality assurance systems with scale-up can mean that increasing quantity does not necessarily compromise quality. Nguyen and colleagues [44] were able to simultaneously scale-up HIV testing for key populations through community-based services and also prevent misdiagnosis by using a valid testing algorithm and quality assessment tools and systems. Bock and colleagues demonstrate that through assessing testing quality and identifying serious testing issues, by following up and implementing retraining, additional supervision, the use of a second reader for RDT results and retesting prior to ART, testing quality improved and errors became infrequent [21].

## Can we have quantity and quality?

Correct HIV test results are one of the WHO “5Cs” and a guiding principle to the delivery of HIV testing services worldwide. Achieving the UN 90-90-90 targets is key to the global public health agenda; however, achieving these goals while also meeting quality testing standards has proven difficult. Continued expansion of HIV testing services and treatment has tremendous individual and public health benefits, but must include accurate diagnosis. Now that ART will be offered to all people with HIV immediately after diagnosis, preventing and addressing misdiagnosis is of paramount importance. Every effort to prevent and address misdiagnosis if and when it occurs must be made alongside the scale-up of HIV testing services.

Communicating and coping with uncertainty in any health-related test results is difficult for healthcare providers and patients alike. On occasion, it may not be possible to deliver an HIV diagnosis on the same day, and further testing after a period of time will be needed. This message must be understood and conveyed by testing providers to their clients. Developing community messaging around the limitations of testing in certain contexts, despite their high accuracy and reliability, may be beneficial. In particular, messaging around the possibility that some clients may not be able to receive a same day diagnosis and returning for test results will be needed. Furthermore, although uncertainties may occur, misdiagnoses are mostly preventable through quality systems, appropriate algorithm use, retesting prior to ART initiation and follow-up procedures to correct discrepancies should they arise.

Current research demonstrating the benefits of immediate ART, for the individual and to prevent transmission, has led activists, national governments, international donors and the non-governmental community to fund and implement unprecedented efforts to provide treatment for everyone with HIV. We now need the same level of activism and global commitment to insist on accuracy of HIV testing.
